# Editorial: Recent Advances in *Drosophila* Cellular and Humoral Innate Immunity

**DOI:** 10.3389/fimmu.2020.598618

**Published:** 2020-10-02

**Authors:** Laura Vesala, Dan Hultmark, Susanna Valanne

**Affiliations:** ^1^ Faculty of Medicine and Health Technology, Tampere University, Tampere, Finland; ^2^ Department of Molecular Biology, Umeå University, Umeå, Sweden

**Keywords:** *Drosophila melanogaster*, innate immunity, cellular immunity, humoral immunity, model for human diseases, metabolism, bacteria, host-pathogen interaction

We are pleased to present our Research Topic for *Frontiers in Immunology*, focusing on “Recent Advances in *Drosophila* Cellular and Humoral Innate Immunity”. This collection of articles is comprised of primary research articles and reviews of the current literature by *Drosophila* immunity experts. Therefore, the papers feature progress in our understanding of the *Drosophila melanogaster* immune responses against a wide variety of pathogens, covering bacteria, viruses and parasites.

In many insects, bacterial infection induces the production of bactericidal and fungicidal peptides, such as cecropins, defensins, diptericins, and drosomycins, which accumulate in the hemolymph (1, 2). Besides these humoral effectors, Uttenweiler-Joseph et al. (3) identified several other *Drosophila* immune-induced molecules (DIMs or IMs) in the fly hemolymph. Their functions remained unknown until recently, when it was shown that twelve related IMs, the Bomanins (Boms), form a family that participates in the defense against some fungi and bacteria (4). Now, Lin et al. have characterized a gene called Bombardier (bbd), which regulates the defense mediated by the short-form Boms. Bombardier mutants are defective in their Toll pathway-dependent resistance against pathogens; especially candidacidal activity is completely dependent on Bombardier and the short-form Boms. Furthermore, Cohen et al. have identified a function for two other IMs, renamed Daisho1 and Daisho2, which are specifically involved in the defense against filamentous fungi.

Whereas the pathways regulating bacterial defenses are quite rigorously studied (5, 6), we still have limited knowledge about the antiviral immune defense in *Drosophila*. Best understood is the RNA interference (RNAi) system, which is believed to be the major defense against RNA viruses (7). This system recognizes double-stranded RNA molecules, cuts them into 21-nucleotide siRNAs, and uses them as templates to recognize and destroy viral RNA. Here, Torri et al. have investigated how two key components in this defense, Dicer-2 (Dcr-2) and Argonaute 2 (AGO2), are modulated in response to viral infection. Both factors were rapidly upregulated in virus-infected animals. Interestingly, only protein levels were affected, not mRNA, suggesting that these effects are post-transcriptionally controlled. Moreover, Trammell and Goodman have reviewed the emerging mechanisms of using *Drosophila* as a tool to study arthropod-borne viruses (arboviruses) that pose a great global health risk to humans. RNAi, JAK/STAT pathway and induction of STING-mediated immunity have been shown to be involved. The nutritional status of flies in the *Drosophila* model as well as of the vector mosquitoes appears to be an important factor in antiviral defense: while mosquitoes use an RNAi-dependent response during starvation, the bloodmeal provides the insulin needed to activate a JAK/STAT-dependent response during infection. The authors conclude that *Drosophila* is an invaluable tool to study arboviruses, but because *Drosophila* are not the natural host for these arboviruses, there are also limitations.

*Drosophila* blood cells, the hemocytes, have traditionally been divided into three distinct classes; phagocytic plasmatocytes, melanizing crystal cells and lamellocytes, the larval immune-induced hemocyte type. The currently ongoing burst of hemocyte research has revealed a more complex system, identifying intermediate stages and sub-classes, some of which are likely to have functional significance (8–13). Shim et al. contribute to the growing pool of hemocyte research with their findings of two distinct plasmatocyte populations, with different functions in metabolic regulation and maintaining organismal homeostasis. Iwashita et al. studied the function of Gyc76A, a guanylate cyclase type receptor producing cyclic guanosine monophosphate (cGMP), in immune responses. They show that Gyc76A is required for hemocyte proliferation during bacterial infection, by activation of a small GTPase Ras85D-dependently rather than cGMP-dependently. Gyc76A is also involved in the antimicrobial peptide response, but that effect is elicited *via* cGMP-dependent protein kinase DG2 and components of the Toll pathway. Thus, Gyc76A is involved in regulation of both, humoral and cellular immune responses via different routes.

Infections cause major metabolic adjustments in immune cells and at systemic level, balancing the needs of the immune system and the organismal homeostasis (14–16). Understanding these events has wide implications, and, not surprisingly, they have recently attracted growing attention. Parupalli et al. have now directly investigated how hemocytes affect nutrition and growth in *Drosophila* development. They show that ablation of hemocytes leads to reduced growth and insulin insensitivity, resulting in minute flies. A similar phenotype was seen when the larvae were fed a high sugar diet, but the effect could be rescued by genetically activating the hemocytes. Their experiments demonstrate the intimate crosstalk between hemocytes and fat body in controlling the distribution of nutritional resources.

Mitochondria are the central energy-producing organelles in the eukaryotic cells. In their review, Salminen and Vale address the role of mitochondrial variation in innate immunity. This variation may arise from both, the nuclear and the mitochondrial genomes, and through the mito-nuclear interactions. They discuss how mitochondrial variation affects the Krebs cycle metabolites, production of adenosine triphosphate and reactive oxygen species, and how these factors alter the infection outcomes. They also describe the benefits of utilizing *Drosophila* to study the nuclear variation affecting mitochondrial functions and how to construct cytoplasmic hybrid (cybrid) fly strains to study the mtDNA variation apart from variation arising from the nuclear genome.

Vincent et al. show that *Francisella novida* infection leads to metabolic dysregulation in control flies but not in imd pathway mutant flies. Their data indicate that in the *Drosophila*-*F. novicida* host-pathogen model, imd pathway activation is necessary but not sufficient for the metabolic pathology. Most likely both the host and bacterial factors contribute to the metabolic dysregulation, which is also seen with other infection models. Davoodi and Foley have reviewed the current knowledge of *Vibrio cholerae* pathogenesis in *Drosophila*. *V. cholerae* is a gram-negative pathogen causing pandemic outbreaks of cholera, a diarrheal disease. About three million cases are estimated to occur per year, resulting in 100,000 deaths. *D. melanogaster* has been used for modelling the *V. cholerae* pathogenesis since the foundational study in 2005, where it was shown that continuous feeding of *V. cholerae* to adult flies caused a cholera-like disease resulting in weight loss and rapid death. In the last 15 years, complex interactions between the pathogen, host defense, intestinal microbiome and metabolism have been uncovered.

In order to understand host-pathogen interactions and the underlying causes of infection outcomes, it is important to characterize molecules secreted by the pathogens. Nonaka et al. performed a structural and functional analysis of Monalysin, a pore-forming toxin from the entomopathogenic bacterium *Pseudomonas entomophila.* By purifying the endogenous Monalysin, they confirmed its action as a pore-forming toxin and suggest that it might preferably target cell membranes at curved sites, such as tips of filopodia. Their study adds to knowledge on detailed defense mechanisms against toxins. Kenney et al. set out to investigate the properties of Excreted-Secreted (ES) products from the nematode *Heterorhabditis bacteriophora*. Concentrated ES products were shown to inhibit the activity of *Diptericin* and to also enable the infection of *Drosophila* by *Photorhabdus luminescens*, symbiotic bacteria from the *H. bacteriophora* nematode. It will be interesting to characterize and identify the specific proteins in the secretions—these proteins bear potential for the treatment of diseases caused by excessive immune activation.

Parasitoid wasps, such as those belonging to the genus *Leptopilina*, lay their eggs inside *Drosophila* larvae. Along with the egg, they inject venom containing a variety of proteins and vesicles, thought to facilitate parasitoid growth (17). Wan et al. concentrated on these vesicles, which they named venosomes. They found that these extracellular vesicles function as a transport system to deliver venom factors into the lamellocytes, a hemocyte important in defense against parasitoids in *D. melanogaster*. This is sufficient to impair the lamellocyte function. The authors theorize that the venosome-cell interaction could be seen as a level of host-pathogen interactions.

As emphasized already above, *D. melanogaster* is a plastic model for studies from research on evolutionarily conserved basic cellular mechanisms to complex human diseases. Lin et al. have investigated the pathogenic effect of the mutant Huntingtin protein (mHTT), that causes the Huntington disease in humans, in the *Drosophila* model. It was demonstrated that overexpression of mHTT in the nervous system causes lethality, whereas overexpressing mHTT in an immune-reactive tissue, i.e. hemocytes, did not cause direct lethality but immune dysregulation. Future studies will be needed to clarify the molecular interaction between mHTT and the main *Drosophila* immune signaling pathways as well as suppression of phagocytosis. The role of immunity and inflammation in aging and age-related diseases, i.e. “inflammaging”, is another pressing question, as the mean population age increases (18). Arora and Ligoxygakis have reviewed the current understanding of the role of immunity in age-dependent neurodegeneration in *Drosophila*. Is inflammation the cause or consequence of neurodegeneration? Predisposition to an overactive immunity, e.g. by loss of negative regulation of the Imd pathway or loss of autophagy, causes neurodegeneration. On the other hand, immunity can also be neuroprotective, as Draper, the glial engulfment receptor, is important in preventing the accumulation of amyloid structures found in Alzheimer’s disease -associated phenotypes in *Drosophila*.

Lastly, Belmonte et al. discuss in their review the profound differences between sexes in response to infection. As in humans (19), *D. melanogaster* shows strong sexual differences in immune responses at baseline, upon pathogen challenge and in aging. In their extensive literature search, they found that fewer than 10% of immunological studies on *Drosophila* have only used one sex or have not separated their subjects by sex. What can *Drosophila* teach us about immune dimorphism? Including both sexes in functional and mechanistic studies of *Drosophila* immunity gives a more complete picture on the complexity of immune mechanisms. It also increases our understanding of the fundamental mechanisms underlying innate immunity mechanisms and responses to infection.

The field of *Drosophila* immunity has broadened considerably in recent years and it is now in a very productive and exciting phase. This is nicely illustrated in this collection of articles. We would like to thank all the authors for their contributions to this Research Topic as well as the reviewers for their time and input.

## Author Contributions

All authors contributed to the article and approved the submitted version.

## Funding

The Drosophila work in Tampere University was carried out in the Tampere Drosophila Facility, partly funded by Biocenter Finland. DH was supported by the Swedish Research Council, the Swedish Cancer Society, the Academy of Finland and the Sigrid Juselius Foundation.

## Conflict of Interest

The authors declare that the research was conducted in the absence of any commercial or financial relationships that could be construed as a potential conflict of interest.
